# Analysis of IGH allele content in a sample group of rheumatoid arthritis patients demonstrates unrevealed population heterogeneity

**DOI:** 10.3389/fimmu.2023.1073414

**Published:** 2023-01-31

**Authors:** Uta Hardt, Martin M. Corcoran, Sanjana Narang, Vivianne Malmström, Leonid Padyukov, Gunilla B. Karlsson Hedestam

**Affiliations:** ^1^ Division of Rheumatology, Department of Medicine Solna, Center for Molecular Medicine, Karolinska Institutet, Stockholm, Sweden and Karolinska University Hospital, Stockholm, Sweden; ^2^ Department of Microbiology, Tumor and Cell Biology, Karolinska Institutet, Stockholm, Sweden

**Keywords:** immunoglobulin heavy chain, germline gene variation, haplotyping, recombination signal sequence, polymorphism, rheumatoid arthritis, population genetics

## Abstract

Immunoglobulin heavy chain (IGH) germline gene variations influence the B cell receptor repertoire, with resulting biological consequences such as shaping our response to infections and altering disease susceptibilities. However, the lack of information on polymorphism frequencies in the IGH loci at the population level makes association studies challenging. Here, we genotyped a pilot group of 30 individuals with rheumatoid arthritis (RA) to examine IGH allele content and frequencies in this group. Eight novel IGHV alleles and one novel IGHJ allele were identified in the study. 15 cases were haplotypable using heterozygous IGHJ6 or IGHD anchors. One variant, IGHV4-34*01_S0742, was found in three out of 30 cases and included a single nucleotide change resulting in a non-canonical recombination signal sequence (RSS) heptamer. This variant allele, shown by haplotype analysis to be non-expressed, was also found in three out of 30 healthy controls and matched a single nucleotide polymorphism (SNP) described in the 1000 Genomes Project (1KGP) collection with frequencies that varied between population groups. Our finding of previously unreported alleles in a relatively small group of individuals with RA illustrates the need for baseline information about IG allelic frequencies in targeted study groups in preparation for future analysis of these genes in disease association studies.

## Introduction

The human immune system recognizes an indefinite number of antigenic determinants through the use of antigen receptors on naïve T and B cells (TCRs and BCRs). BCRs are transmembrane-bound immunoglobulin (IG) molecules composed of heavy and light chains, encoded by rearranged variable (V), diversity (D) and joining (J) genes, and a constant gene segment (1). The vast sequence diversity of naive IG heavy chain (IGH) repertoires is mediated by combinatorial recombination of V, D and J genes at recombination signal sequences (RSS). These are characterized by a conserved heptamer at the 5’ end, a 12/23 bp spacer, and a conserved nonamer at the 3’ end. The IG diversity is further increased by non-templated nucleotide insertions and/or trimming of nucleotides at the V-D and D-J junctions (2) during the recombination process, and subsequent somatic hypermutation (SHM) and isotype switching that increases antibody-antigen affinity and function.

The IGH locus in humans contains frequent copy number variations and an abundance of pseudogenes interspersed between highly similar functional genes, resulting in a challenge for genomic sequencing (3–5). Traditional short read sequencing approaches, such as those utilized for the 1000 Genomes Project (1KGP) (6, 7), result in ambiguous assemblies of the IGH region, limiting the ability to accurately identify IG gene variations. Furthermore, for complex genomic regions such as the IGH locus, high coverage genomic sequencing is low throughput. Scaling up to large numbers of individuals, such as those in disease cohorts, remains a major challenge. To date, high coverage sequencing of the IGH locus has been reported for a limited number of individuals (8, 9), but there are ongoing studies to extend this analysis for over 100 individuals (10).

In the recent years, the development of next generation sequencing (NGS) approaches that allow sufficient sequencing length and depth has enabled opportunities to infer germline alleles from full-length V(D)J sequences using tools such as IgDiscover (11), TIgGER (12), Partis (13, 14) or IMPre (15) to determine germline IGHV and IGHJ sequences at an individual level. NGS-based immune repertoire sequencing is high throughput offering possibilities to define allele frequencies in larger groups of individuals and enables the application of inferred haplotype analysis to reveal gene duplication and structural variation (16).

The Epidemiological Investigation of Rheumatoid Arthritis (EIRA) study is a population-based case-control study based on incident cases of rheumatoid arthritis in Sweden. EIRA comprises adult individuals in areas from southern and central Sweden from May 1996 and onwards. Cases were recruited through rheumatology clinics in the study area (17). Controls were randomly selected from the population registry shortly after case identification and were matched on age, sex and residential area. 96% of participating cases and 60% of participating controls provided blood samples. Cases and controls were invited to answer an extensive questionnaire. To date, the study population consists of several thousand cases and controls. Here, to examine IGH allelic variation in a pilot group comprising 30 individuals belonging to the EIRA study, in preparation for larger association studies, we generated IgM libraries and applied the germline inference tool IgDiscover to each case.

Within the 30 case dataset, we classified the IGH germline allelic genotype of each case, thereby enabling the identification of novel allelic variants and biased allelic expression in the IgM repertoire by inferred haplotype analysis. Variants identified were validated using haplotyping and Sanger sequencing. Most notably, we discovered a novel IGHV4-34 gene variant, which was present in 10% of the cases and that could be validated by restriction fragment length polymorphism analysis. Of critical importance, a set of 30 control cases was therefore included in the restriction fragment length polymorphism analysis to delineate the frequency of this allele in healthy individuals of the same population group. The results obtained here expand our knowledge of IGH gene diversity and highlight the importance of extended and population specific profiling of the baseline content and frequencies of IG alleles prior to performing disease association studies. Any such population based allelic frequency bias has the potential to confound association studies and the EIRA study set of patient samples and matched controls provides an ideal opportunity to extend our findings.

## Results

### Personalized genotyping of IGHV alleles

**Figure 1:**
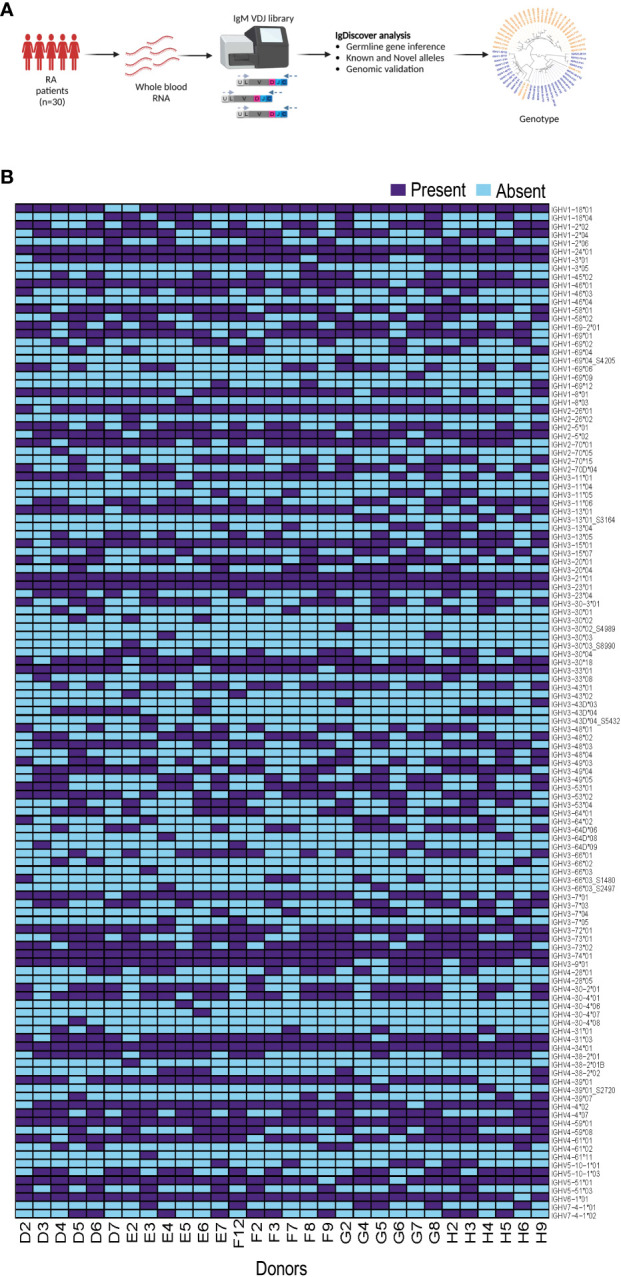
Production of immunoglobulin heavy chain genotype information from 30 individuals. **(A)** The flowchart shows the process from whole blood RNA isolation, IgM library preparation and subsequent IgDiscover analysis. **(B)** Heatmap showing expressed IGHV alleles for all 30 individuals.

We generated IgM libraries from 30 female individuals without genetic indication of non-European ethnicity from the EIRA study using whole blood RNA as the input material. The libraries were generated by reverse transcription PCR using an IgM gene specific primer, followed by multiplex PCR using IGHV leader-specific primers, and subsequent index PCR to add the Illumina adapters, as previously described (18). After sequencing using the Illumina MiSeq platform, we used the IgDiscover germline inference tool to infer individualized genotypes of each case (**Figure 1A**). We inferred eight novel IGHV alleles among the 30 individuals as indicated in the allelic heatmap with an underscore and suffix S number. Four novel alleles, IGHV1-69*04_S4205, IGHV3-30*02_S4989, IGHV3-30*03_S8990 and IGHV3-43D*04_S5432, were present in one individual, two novel alleles, IGHV3-66*03_S2497 and IGHV4-39*01_S2720, were present in two individuals. IGHV3-13*01_S3164 was present in four individuals and IGHV3-66*03_S1480 was present in five individuals. The inferred expressed IGHV alleles for all 30 individuals are shown as a heatmap in **Figure 1B**.

### Haplotyping by IGHJ6 revealing heterozygosity status

**Figure 2:**
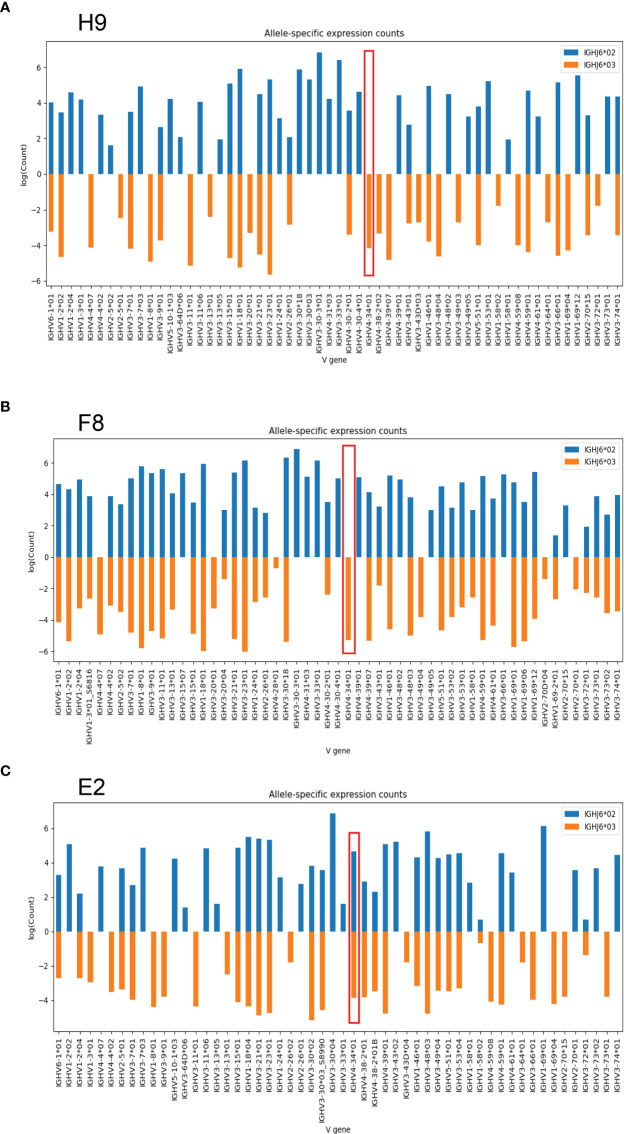
Haplotype analysis of two individuals based on IGHJ6 heterozygosity. **(A)** J6-anchored haplotype analysis of individual H9 reveals IGHV4-34 hemizygosity (red box). **(B)** J6-anchored haplotype analysis of individual F8 reveals IGHV4-34 hemizygosity (red box). **(C)** Reference haplotype from individual E2 reveals IGHV4-34 homozygosity.

As previously shown, approximately 25-30 percent of humans are heterozygous for the IGHJ6 gene (19). Consistent with this, nine of the 30 individuals included in this study were found to be heterozygous with the presence of both IGHJ6*02 and IGHJ6*03. Since VDJ recombination occurs locally along a single chromosomal strand, IGHJ6 heterozygosity can be used to anchor IGHV alleles to a specific haplotype. In this manner IGHV alleles can be revealed as homozygous, heterozygous, duplicated or deleted (16, 20, 21). In examining haplotype plots for the haplotypable cases studied here, we observed an unexpected hemizygosity for IGHV4-34 in two individuals, H9 and F8. In both cases, we could map one allele, IGHV4-34*01, to one chromosome while expression of IGHV4-34 was absent from the other chromosome (**Figures 2A, B**). In the reference haplotype from individual E2, there is evident IGHV4-34*01 homozygosity (**Figure 2C**).

### RSS SNP variant associating with decreased IGHV4-34 expression

IGHV4-34*01 is a very common allele of IGHV4-34, a gene that shows low levels of allelic variation in previous inference studies (19). Absence of an expressed allele on one chromosome can be explained by either a genomic deletion of IGHV4-34 on that chromosome or by mechanisms that interfere with either the recombination, expression or stability of the variant allele.

Genomic amplification and Sanger sequence analysis of IGHV4-34 in both cases with monoallelic expression (H9 and F8) resulted in the identification of IGHV4-34 heterozygosity, with two allelic variants found in both individuals. In each case, these variants included the common IGHV4-34*01 allele, and a novel variant, IGHV4-34*01_S0742, that shared 100% sequence identity to IGHV4-34*01 across the entire V sequence, but contained a single nucleotide polymorphism (SNP) within the second position of the seven bp RSS heptamer sequence, resulting in the non-canonical heptamer sequence, CTCAGTG.

**Figure 3:**
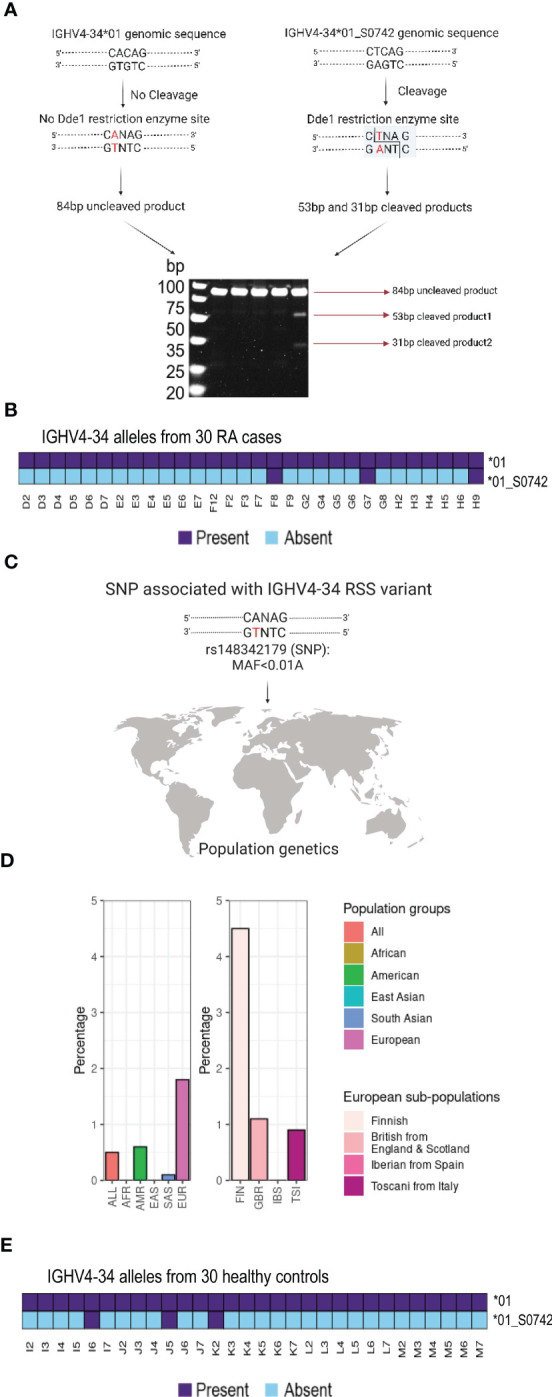
RSS variant analysis in the study group and in populations **(A)** Scheme of the restriction fragment length polymorphism is shown. **(B)** Three RA individuals contain the RSS variant of the IGHV4-34 gene (IGHV4-34*01_S0742). **(C)** Schematic overview of the SNP number associated with the polymorphism **(D)** Data from the 1000 Genomes Project reveals that the coding minus strand has a T/A variant in SNP rs148342179 that is especially frequent in the Finnish (FIN) population. **(E)** Three healthy controls contain the RSS variant of the IGHV4-34 gene (IGHV4-34*01_S0742).

Since 21 of the 30 cases could not be J6 haplotyped we could not investigate if there were additional cases that were heterozygous for the IGHV4-34 RSS variant allele using the haplotyping approach. However, the IGHV4-34 RSS variation results in the introduction of a restriction site for the enzyme DdeI that recognizes the target sequence CTNAG that is absent from the IGHV4-34*01 RSS. We therefore PCR amplified an 84 bp segment spanning the polymorphism using genomic DNA from all 30 cases. These were analysed by restriction fragment length polymorphism analysis (RFLP), which allowed us to identify heterozygous cases containing this allelic variant. DdeI digestion of an 84bp amplicon produced three diagnostic bands of 84, 53 and 31bp in heterozygous cases containing the variant (**Figure 3A**), while individuals without the variant yield only the undigested 84 bp product. We found that the two haplotyped individuals, F8 and H9, as well as one additional individual, G7, produced the diagnostic bands (**Figure 3B**; **Supplemental Figure 1** and **Supplemental Tables 1, 2**), demonstrating a prevalence of the RSS variant allele, IGHV4-34*01_S0742, in 10 percent of cases in our study. Updated IGHV genotypes of H9, F8 and G7 including the non-functional IGHV4-34*01_S0742 allele are shown in **Supplemental Figure 2**. The non-canonical CTCAGTG heptamer is consistent with an interference in effective VDJ recombination, which explains the IGHV4-34 hemizygosity in these individuals.

### Population frequency of IGHV4-34*01_S0742

The IGHV4-34*01_S0742 RSS variant nucleotide was consistent with SNP rs148342179 (A/T) (**Figure 3C**). This SNP was found to be present in 0.5 percent of all samples of the 1000 Genomes Project (ALL) and in 1.8 percent of the European samples (EUR). It was not found in either the African (AFR) or East Asian samples (EAS) and was only present at low frequency in the other populations such as the American (AMR: 0.6 percent) and the South Asian (SAS: 0.1 percent). However, rs148342179 is present at much higher frequency among Finnish individuals (FIN: 4.5 percent) within the European population (**Figure 3D**). To determine the frequency of this allele in a matched control population, we performed genomic PCR and DdeI restriction digestion on a set of 30 control samples. We found the variant was present in three control samples, I6, J5 and K2 and we validated the presence of the IGHV4-34*01_S0742 allele in all three individuals by targeted genomic PCR and Sanger sequencing (**Figure 3E** and **Supplemental Tables 1, 2**).

### Identifying a novel IGHJ6 allele as an anchor for haplotyping

**Figure 4:**
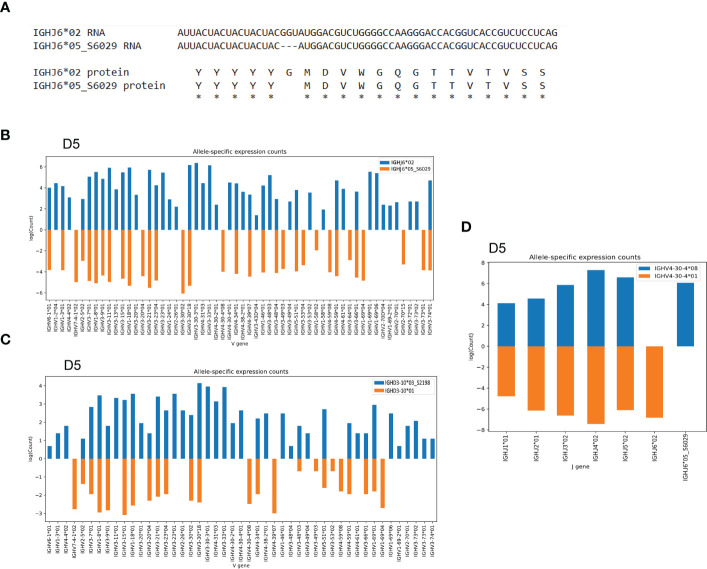
Novel IGHJ6 allele as anchor for haplotyping **(A)** The alignment shows the deletion of a base triplet in the novel IGHJ6*05_S6029 results in a deletion of a glycine residue at protein level. **(B)** The novel IGHJ6*05_S6029 variant in combination with the regular IGHJ6*02 allele can be used for IGHV gene haplotyping in the D5 individual. **(C)** Case D5 can be haplotyped based on IGHD3-10 gene heterozygosity. **(D)** The J6 alleles are haplotypable using heterozygous IGHV4-30-4 alleles as anchors, showing the clear separation of the IGHJ6*05_S6029 allele to a different chromosome compared to the IGHJ6*02 allele.

In addition to the novel IGHV4-34 allele, we found a novel IGHJ6 allele, IGHJ6*05_S6029, in individual D5. This variant J allele was characterized by the deletion of a triplet base GGT, which we validated using targeted genomic PCR and Sanger sequencing. This deletion is consistent with a SNP variant, rs74454466 and results in a deletion of a single glycine at the protein level (**Figure 4A** and **Supplemental Table 2**). The presence of this variant IGHJ6 allele provided an additional heterozygous J6 anchor, enabling IGHV haplotype analysis of D5 (**Figure 4B**), thereby allowing J6-anchored haplotype analysis of a total of 10 of the 30 individuals. In addition to the anchoring by J6 gene heterozygosity, it is possible to use IGHD3-10 or IGHD2-21 heterozygosity to infer V gene haplotypes. In the current study, this allowed haplotyping of five additional individuals of the 30 cases. In case D5, we identified a novel IGHD3-10 allele, IGHD3-10*03_S2198, providing two heterozygous anchors in this individual. This allowed a direct comparison of using heterozygous J and D genes for haplotyping, illustrating that the use of J anchors is preferred as this gives higher sequence counts for each V allele (**Figures 4B, C**). Finally, to validate the presence of the novel IGHJ6*05_S6029 allele, we used a heterozygous V gene, IGHV4-30-4*01/IGHV4-30-4*08, to haplotype J genes in D5, which clearly demonstrated the presence of IGHJ6*02 and IGHJ6*05_S6029 on the two separate chromosomes (**Figure 4D**).

### Common structural variation

**Figure 5:**
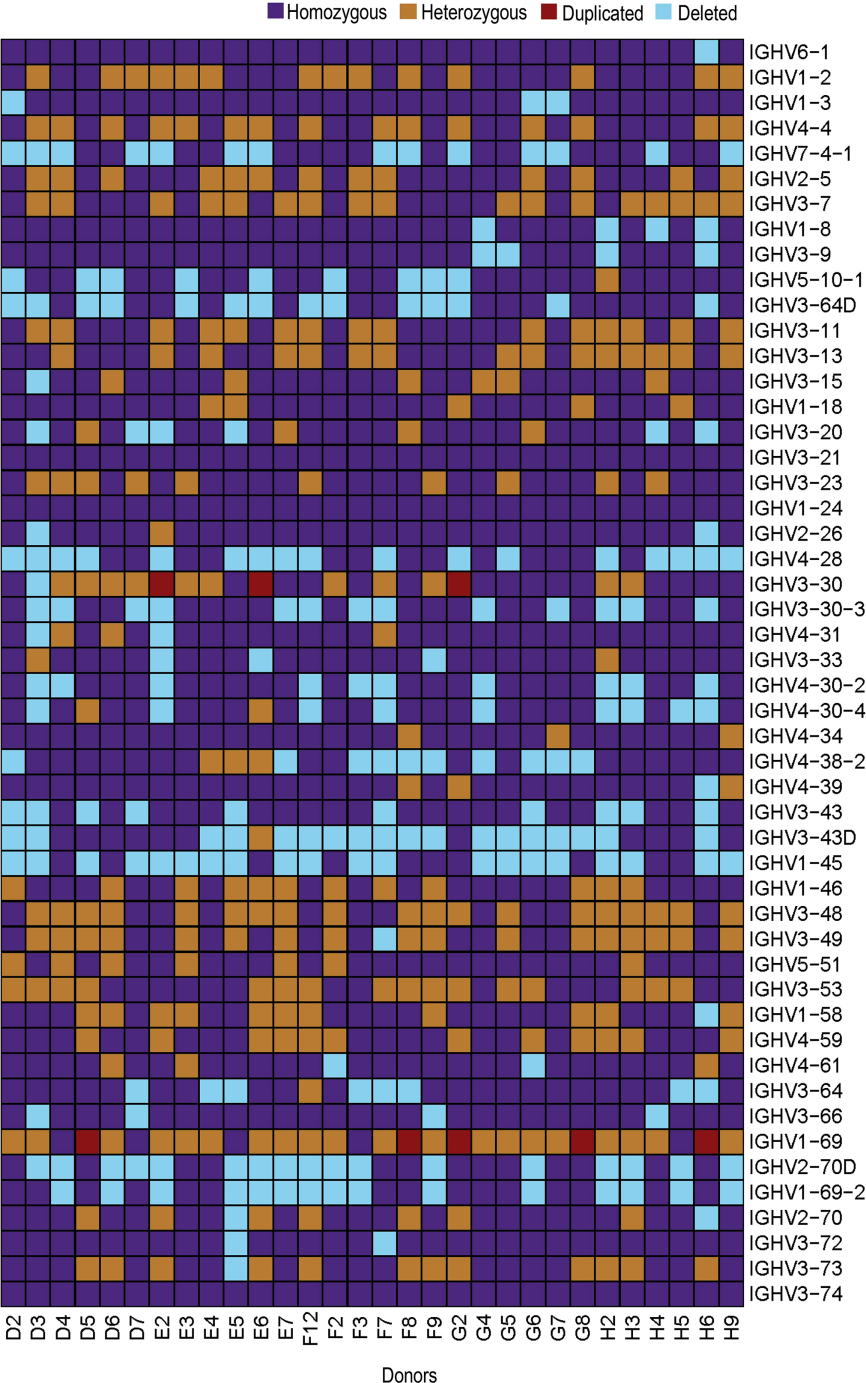
Heatmap showing IGHV genes for all 30 individuals. IGHV genes are represented on y-axis in chromosomal order with color-key on the top-right.

At the gene level, several common structural variations were identified in the 30 individuals. Duplication of the IGHV3-30 gene was apparent in three individuals and of the IGHV1-69 gene in five individuals. The complete set of IGHV3-30, IGHV3-30-3, IGHV4-31, IGHV3-33, IGHV4-30-2 and IGHV4-30-4 genes was present in 14/30 individuals and the genes for IGHV2-70D and IGHV1-69-2 were present in 13/30 individuals. The IGHV4-38-2 gene was absent in 10 individuals, while the IGHV3-43D gene was absent in 18 individuals. In addition, the genes for IGHV1-8 and IGHV3-9 were present in a homozygous state in 25/30 individuals, while the genes for IGHV3-64D and IGHV5-10-1 were present in homozygous state only in 15/30 individuals (19). (**Figure 5**). Overall, our analyses demonstrate an extensive variation in the IGH locus, at both structural and allelic levels.

## Discussion

Here, we defined the IGH genotype of 30 individuals from the EIRA study. We found a novel IGHV4-34*01_S0742 allele, which was present in 10 percent of the individuals. This allele was characterized by a SNP, rs148342179, in the RSS region, which interfered with successful recombination, resulting in hemizygous expression of IGHV4-34. The location of the variant T nucleotide at position 2 of the RSS heptamer deviates from the canonical CA sequence described in previous analyses of RSS functionality and may therefore have a major inhibitory effect on recombination (22), as reflected by the absence of expression of the allele in cases H9 and F8. In addition to the inferred haplotype analysis, which shows a hemizygous loss of a functional IGHV4-34 in heterozygous cases, it is important to note that an A to T change in the RSS heptamer has not been described for any functional RSS heptamer in humans or other species. The starting CA dinucleotide is believed to be critical for the recombination process as shown by Kim et al. (23), with CA facilitating reduced base-stacking and enabling bending of the DNA helix during the recombination process. Consistently, Hu et al. (24) report 107 cryptic RSS sites, all of which require a CAC triplet at the beginning of the heptamer RSS sequence. Finally, Hoolehan et al. (25) recently showed that the CAC triplet is crucial feature in functional RSS heptamers, with all functional non-canonical RSS heptamer sequences showing nucleotide variation solely in the final four nucleotides.

Although the population origin was not recorded within the EIRA study, we know that the participants were collected in the middle and southern parts of Sweden. Data from the 1000 Genomes Project show that SNP rs148342179 found in our cases was present at 4.5 percent in the Finnish population. The population frequency of IGHV4-34*01_S0742 identified in our study can be expected to result in around one percent of the Finnish/Scandinavian population being homozygous for this RSS variant, thereby resulting in the full absence of IGHV4-34 expression in the immunoglobulin repertoires in these individuals. Similarly, homozygous deletions of the IGHV3-30/IGHV4-31 region (hv3005) were found to be enriched in RA patients (26) and SLE patients compared to ethnically matched healthy individuals of Korean (27) or Caucasian (28) ethnicity. This deletion has also been associated with susceptibility to chronic idiopathic thrombocytopenic purpura in Caucasians (29).

The IGHV4-34 gene has previously been shown to be associated with autoimmunity (30), but the specificities responsible for this remain unclear. IGHV4-34 is highly used in both the IgM and IgG repertoire (31), and has been identified in studies of autoreactivity towards type I blood antigens (32). In particular, autoreactive antibodies encoded by the IGHV4-34 gene have been shown to be raised in patients with systemic lupus erythematosus (SLE) using the rat monoclonal antibody 9G4 (33) that has been claimed to recognize human IGHV4-34. In a repertoire analysis study, Bashford-Rogers et al. (34) found increased usage of IGHV4-34 in 10 patients with SLE of mixed Caucasian and Asian ethnicity, 11 patients with eosinophilic granulomatosis with polyangiitis of mainly northern European ancestry and 23 patients with Crohn’s disease of mainly northern European ancestry compared to healthy individuals of mainly northern European ancestry. In SLE, there is a plethora of autoreactivities described, however the over-representation of IGHV4-34 among SLE clonal expansions (35) is insufficient to conclusively prove specific autoreactivity of that germline gene, particularly in the context of other diseases. The IGHV4-34*01 allele contains a germline-encoded Asn-X-Ser/Thr motif in its CDR2 region, which allows N-linked glycosylation at this site (36). High levels of SHM-introduced variable domain glycans have been associated with autoantibodies in rheumatoid arthritis (37). At the same time, it has been shown, that an antibody produced by a self-reactive B cell had reduced capacity of autoantigen binding, when N-linked glycosylation was introduced (38). However, we note that in this particular RA group analyzed, the frequency of the IGHV4-34*01_S0742 variant exactly matches that of the control group. While the allele may have functional significance within the population at large, particularly in the case of homozygosity, we did not find a clear signal that it was relevant to the RA group in the current study.

A similar observation to the IGHV4-34*01_S0742 allele, where an RSS polymorphism affected the V gene usage, was reported for a variant kappa V gene, IGKV2-29D, enriched in a Native American population. In that study the variant was associated with *Haemophilus influenzae* type b susceptibility (39, 40). Of critical importance to this study was the observation that the frequency of the heterozygous IGHV4-34 variant in the patient group was identical to that found in the matched control group. The frequencies of IGHV4-34*01_S0742 in both the Swedish EIRA patient samples and in the healthy controls studied here are consistent with 1000 Genomes population data showing SNP rs148342179 is found at highest frequencies in the Finnish population set, a region geographically and historically closely linked to Sweden. It is interesting to note that the haplotypable cases H9 and F8 share an identical string of 14 alleles, from IGHV3-30-3*01 to IGHV5-51*01, that encompasses the non-functional IGHV4-34*01_S0742 allele, indicating the possibility that the mutation may be historically recent. Further studies may reveal how common this shared segment is within this and other populations.

SNP arrays and whole genome sequencing enabling association studies have revolutionized the field of complex disease genetics. Genome wide association studies have implicated causal genes and mechanisms, drug targets, disease biomarkers and risk prediction. There are more than 150 risk loci reported in genome wide association studies of rheumatoid arthritis patients from different populations (41). The class II human leukocyte antigen (HLA) loci show a very strong association with rheumatoid arthritis. Together with efficacy of anti-CD20 therapy, rituximab (42), these data indicate a role for T and B cells in rheumatoid arthritis pathology. Genotyping the complex IGHV locus with the aim of identifying disease associated variation is challenging. The process necessitates the ability to produce accurate and comprehensive genotypes encompassing genes that are utilized at both low and high frequency in the B cell repertoire. Structural variations due to gene deletion or duplication events, in addition novel alleles that may be present in the population (18) should also be identifiable. High variation in the IG loci may introduce confounding problems in association studies if the case and control groups are not population-stratified (43) since the goal is to identify disease related polymorphisms that are present at different frequencies in the disease group compared to the control group. The observation that IG genes display great population variation has been discussed with the respect to limited sampling groups currently represented by databases (44).

Notably, genome wide association studies currently lack high quality information for the highly complex immunoglobulin heavy and light chain loci. As immunoglobulins play an important role as B cell effector molecules in rheumatoid arthritis, these loci may equally be a source of genetic variance within the general population that contributes to disease susceptibility and are therefore an important target for disease association studies. An example of such variation was identified in the indigenous populations of the South Pacific, who expose a high burden of rheumatic heart disease. The allele IGHV4-61*02 is associated with an increased risk of rheumatic heart disease (45) in that population set. Identification of the functional allelic variation in the IGHV locus is challenging, but feasible using specialized next generation sequencing methods and computational analysis, as applied in our studies.

Previous studies on antibody repertoires in rheumatoid arthritis are to our knowledge mostly limited to studies of heavy chain CDR3 sequences derived from comparatively small libraries constructed with a limited number of IGHV primers (46). In our studies, we use a 5’ MTPX primer set that was previously shown to capture a comprehensive set of IGHV genes and we sequence full-length VDJ transcripts and determine IG genotypes in each study participant (18). Individualized genotyping of individuals provides the basis for analysing IGHV germline gene variation and expressed repertoires in any disease, including rheumatoid arthritis. However, interpreting IGHV allelic variation can be challenging since there may be population-based variations in allelic frequencies. Identification of immunoglobulin gene polymorphisms in a disease study group, even at high frequency, should not be assumed to be disease related if that frequency matches that of the population from which the study group is drawn.

The current study was not designed or powered for association analysis; however, this can be performed with the larger EIRA study with matching control samples already available (47). Association studies for immunoglobulin alleles are so far limited to studies that are reviewed elsewhere (48, 49); thus the impact of this variation on disease risks is insufficiently investigated (50, 51). The results of our pilot investigation demonstrates that population-based genetic variance of IG alleles is likely to be common (45, 52–54). Without adequate information of the expected frequencies of immunoglobulin alleles in the population, erroneous associations may be identified. Likewise, real associations may become apparent only when accurate information of allelic frequencies in the target population is well established.

## Materials and methods

### Experimental design

We collected whole blood samples in PAXgene tubes (Qiagen) from 30 rheumatoid arthritis (RA) patients and genomic DNA samples from 30 healthy controls. The inclusion criteria were recruitment within the EIRA (Epidemiological Investigation of Rheumatoid Arthritis) study (55), no indication from the Immunochip Array genotyping (Illumina) for non-European ancestry. Whole blood RNA (extracted with PAXgene Blood miRNA Kit, PreAnalytiX, Qiagen) was used to prepare cDNA for subsequent construction of IgM libraries (18) to infer Immunoglobulin heavy chain genotypes. Sampled DNA was used to validate inferred novel variants by RFLP and Sanger sequencing.

### Patients

All RA patients and healthy controls were recruited as part of the EIRA study under ethics permits #1023-96 and #2006/476-31/4 obtained from Regionala Etikprövningsnämnden, Stockholm. This study comprises cases and control subjects from the middle and southern parts of Sweden. All samples were taken in hospital-based or privately run rheumatology units in the study area in accordance with the Helsinki Declaration and written informed consent was given by each patient before entering the study. In the current study, we included 30 female RA patients comprising 10 shared epitope-negative (SE negative) anti-citrullinated protein antibody-negative (ACPA negative) individuals with a mean age of 62.5 years, eight SE positive ACPA negative individuals with a mean age of 57.4 years and 12 SE positive ACPA positive individuals with a mean age of 58.7 years.

### Library preparation

IgM libraries were prepared according a previously published protocol (18). In brief, 200 ng of whole blood mRNA was reverse transcribed using the Sensiscript Reverse Transcription kit (Qiagen) and reverse gene specific primer with a unique molecular identifier (UMI) and a universal reverse amplification sequence. 2 µl of purified (Qiagen MinElute PCR purification kit) cDNA was amplified using the universal reverse primer and the chain-specific 5’ forward leader primer mix, using the KAPA HiFi Hotstart Ready Mix (Roche). The product of around 480bp was gel purified (Qiagen MinElute Gel Extraction kit). 5 to 10ng of the gel-purified product were used for the indexing PCR, as detailed previously (18). The forward indexing primer P5_R1 and the reverse indexing primer P7_R2_I1-27 were added in 10 cycle PCR reaction using the KAPA HiFi Hotstart Ready Mix (Roche). The final libraries were purified and quantified according to Illumina’s manufacturer’s instructions. The Illumina Version 3 (2x300bp) sequencing kit was employed for sequencing the libraries with the addition of 13% PhiX174 DNA (12pM) as positive control.

### Computational analysis

Library analysis was performed using the IgDiscover version 0.12.4 with default settings. IgDiscover pre-processed the libraries for quality control and subsequently performed expression analysis and generated individualized databases. The IMGT reference database (May 2019) was used (56), with the addition of some recently described new alleles (57). The databases were aligned using CLUSTAL W (58) and the trees were plotted using FigTree (version 1.4.4). Haplotypes were generated using the plotalleles module of IgDiscover with a chromosomal filter of 25%.

### Software

Heatmaps and 1000 Genomes Project data were plotted with R (version 3.6.3) using R studio (version 1.2.1335). In particular, we used tidyverse (version 1.3.0), cowplot (version1.1.1) and gplots (version 3.1.3) packages.

### Restriction fragment length polymorphism

84bp long genomic DNA around the polymorphism was amplified using specific primers designed using BLAT (59) (UCSC genome browser, **Supplemental Table 3**). Amplicons were digested by DdeI (Thermo Fisher Scientific) for 4h at 37°C. Samples were run on a TBE 20% polyacrylamide gel (Invitrogen) at 100V for 3.5h and stained with SYBR Green I nucleic acid gel stain (Thermo Fisher Scientific) in TE buffer (10mM Tris, 1mM EDTA, pH8) for 40 min while shaking. Images were taken on a Biorad Geldoc instrument.

### Genomic validation

Primers encompassing the IGHV4-34 gene and the IGHJ6 gene were designed using BLAT (UCSC genome browser) to validate the presence of novel alleles (**Supplemental Table 3**). 10ng of genomic DNA template were amplified using KAPA Hifi Hotstart Ready Mix (Roche). The product was gel purified (Qiagen MinElute Gel Extractration kit) and ligated to CloneJet pJET 1.2 vector (Thermo Scientific). 1 to 2µl of ligation product were transformed in XL10-Gold Ultracompetent Cells (Agilent) following manufacturer’s instructions. Transformed Cells were grown overnight on a 100 mg/ml ampicillin LB agarose plate before colony screening. IGHV4-34 gene transformed colonies were screened using the RFLP primers, IGHJ6 gene transformed colonies were screened for inserts. Positive colonies were grown in LB medium overnight. Bacterial cultures were purified with GeneJET Plasmid Miniprep Kit (Thermo Scientific) and Sanger sequenced (Genewiz). Sanger sequences of validated alleles have been deposited in the GenBank under accession numbers OL807662 (IGHV4-34*01_S0742) and OL807663 (IGHJ6*05_S6029).

## Data availability statement

The datasets presented in this study can be found in online repositories. The names of the repository/repositories and accession number(s) can be found below: https://figshare.com/, 10.17044/scilifelab.21316677 https://www.ncbi.nlm.nih.gov/genbank/, OL807662, OL807663.

## Ethics statement

The studies involving human participants were reviewed and approved by Regionala Etikprövningsnämnden, Stockholm. The patients/participants provided their written informed consent to participate in this study.

## Author contributions

UH, SN and MC designed and performed the experiments. UH, MC, SN, VM, LP and GKH analysed the data. UH, MC and GKH wrote the paper. All authors contributed to the article and approved the submitted version.

## Supplementary material

Supplementary Figure 1 Restriction digestion of a PCR product comprising the variant position identified in IGHV4-34*01_S0742 (rs148342179) shows the diagnostic 53 and 31bp bands in individuals F8 and G7, and H9.

Supplementary Figure 2 IGHV genotypes of H9, F8 and G7 including the IGHV4-34*01_S0742 allele.
