# Supplementary figures and images for: Analysis of IGH allele content in a sample group of rheumatoid arthritis patients demonstrates unrevealed population heterogeneity
Source: Front Immunol. 2023 Jan 31;14:1073414. doi: 10.3389/fimmu.2023.1073414 (PMC9927645; doi:10.3389/fimmu.2023.1073414)

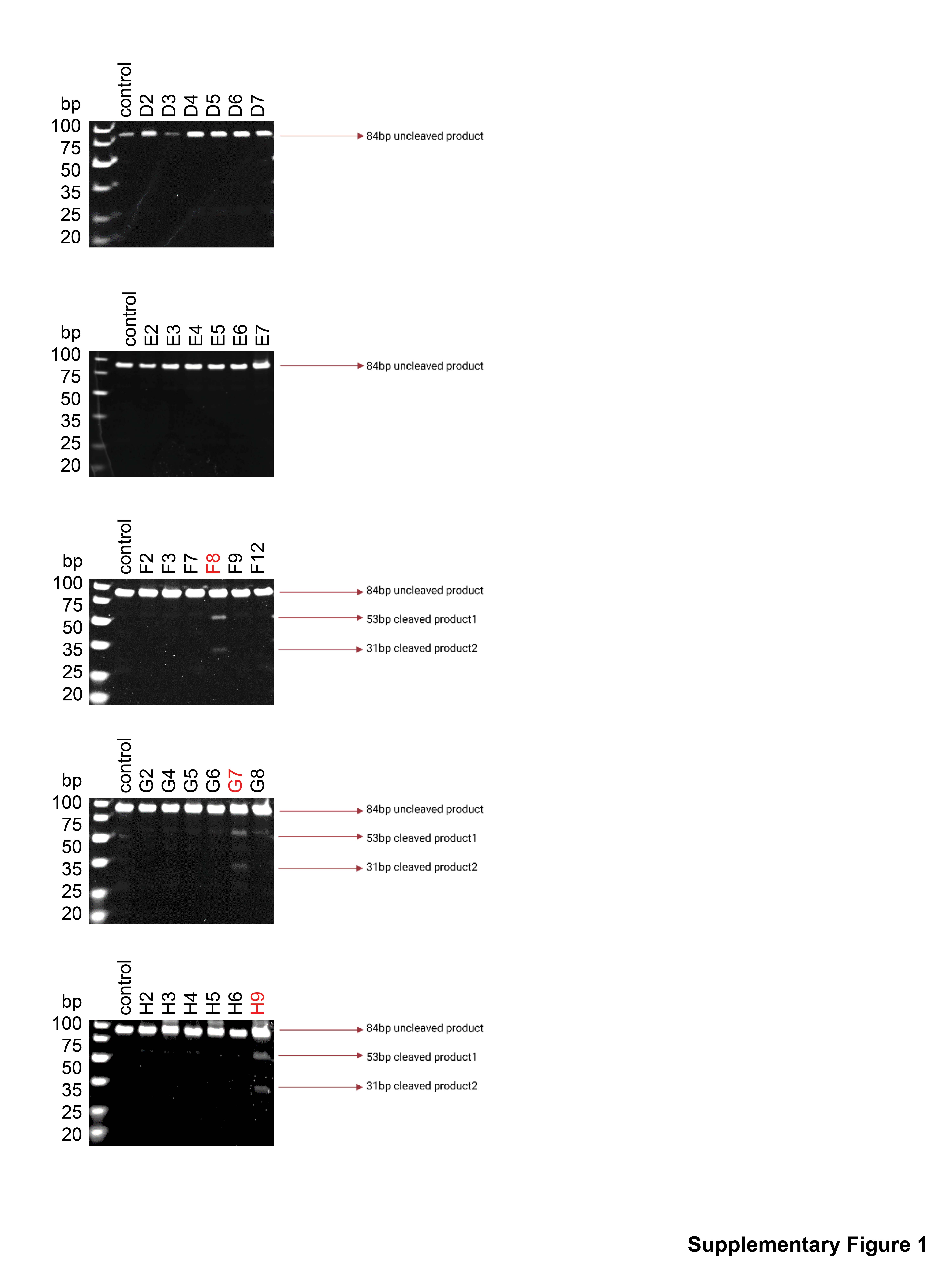

Supplement: Supplementary Figure 1 — Restriction digestion of a PCR product comprising the variant position identified in IGHV4-34*01_S0742 (rs148342179) shows the diagnostic 53 and 31bp bands in individuals F8 and G7, and H9. [file Image_1.jpeg]

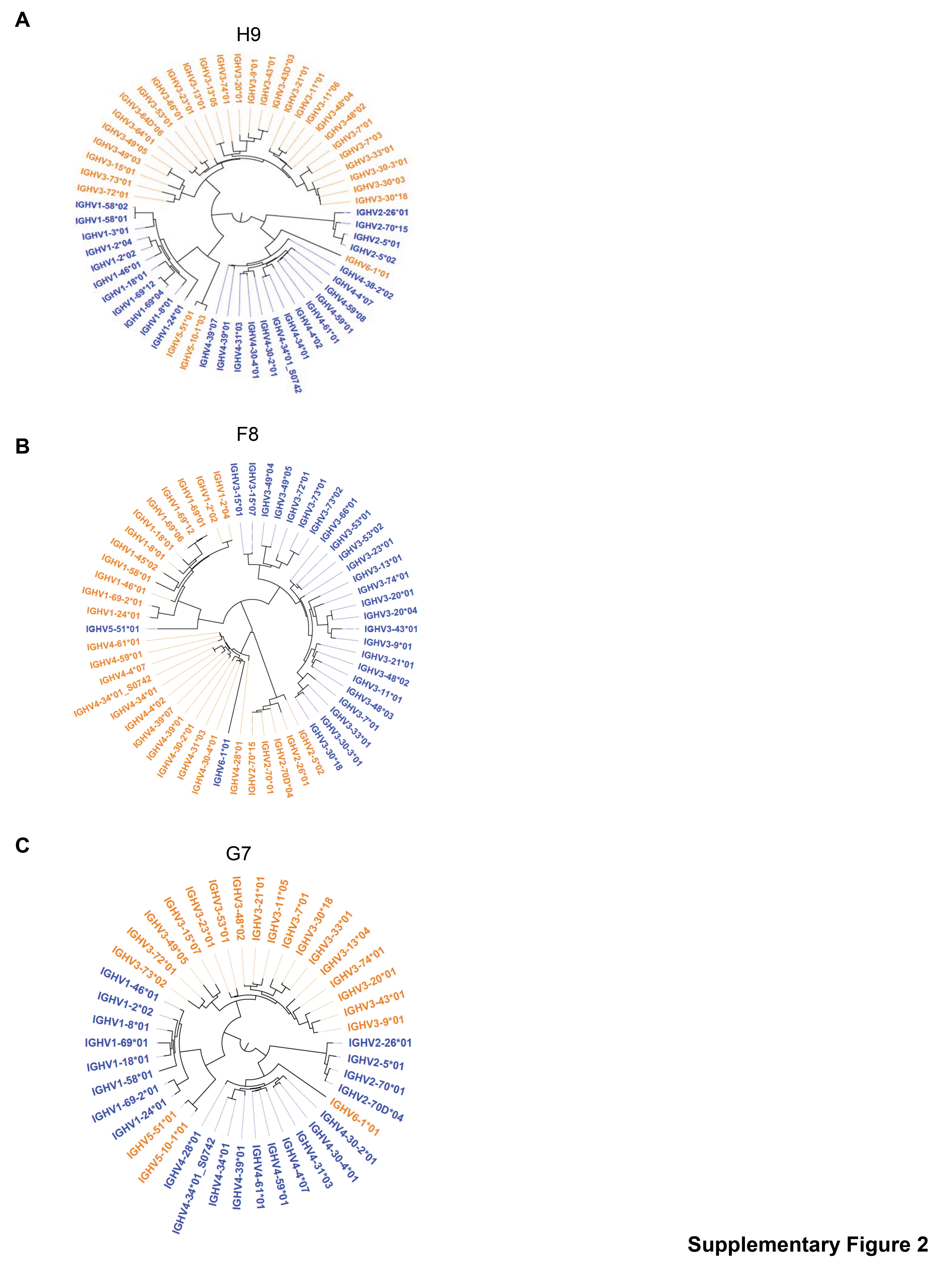

Supplement: Supplementary Figure 2 — IGHV genotypes of H9, F8 and G7 including the IGHV4-34*01_S0742 allele. [file Image_2.jpeg]
