# Supplementary material for: Analysis of IGH allele content in a sample group of rheumatoid arthritis patients demonstrates unrevealed population heterogeneity
Source: Front Immunol. 2023 Jan 31;14:1073414. doi: 10.3389/fimmu.2023.1073414 (PMC9927645; doi:10.3389/fimmu.2023.1073414)
Supplement: Supplementary file 3 [file Table_1.docx]

**Supplemental Table 1.**

| Case | group | PCR + DdeI digest | Sanger validation | |
| --- | --- | --- | --- | --- |
| F8 | RA | heterozygous | Yes |  |
| G7 | RA | heterozygous | Yes |  |
| H9 | RA | heterozygous | Yes |  |
| I6 | control | heterozygous | Yes |  |
| J5 | control | heterozygous | Yes |  |
| K2 | control | heterozygous | Yes |  |
