# Supplementary material for: Analysis of IGH allele content in a sample group of rheumatoid arthritis patients demonstrates unrevealed population heterogeneity
Source: Front Immunol. 2023 Jan 31;14:1073414. doi: 10.3389/fimmu.2023.1073414 (PMC9927645; doi:10.3389/fimmu.2023.1073414)
Supplement: Supplementary file 4 [file Table_2.docx]

**Supplemental Table 2**

| **IGHV Allele** | **Leader1** | **Leader2** | **V Sequence** | **RSS** | **23 bp Spacer** | **Nonamer** |
| --- | --- | --- | --- | --- | --- | --- |
| IGHV4-34*01_S0742 | ATGAAACACCTGTGGTTCTTCCTCCTCCTGGTGGCAGCTCCCAGAT | GGGTCCTGTCC | CAGGTGCAGCTACAGCAGTGGGGCGCAGGACTGTTGAAGCCTTCGGAGACCCTGTCCCTCACCTGCGCTGTCTATGGTGGGTCCTTCAGTGGTTACTACTGGAGCTGGATCCGCCAGCCCCCAGGGAAGGGGCTGGAGTGGATTGGGGAAATCAATCATAGTGGAAGCACCAACTACAACCCGTCCCTCAAGAGTCGAGTCACCATATCAGTAGACACGTCCAAGAACCAGTTCTCCCTGAAGCTGAGCTCTGTGACCGCCGCGGACACGGCTGTGTATTACTGTGCGAGAGG | CTCAGTG | AGGGGAGGTGAGTGTGAGCCCAG | ACAAAAACC |
|  |  |  |  |  |  |  |
