# Supplementary material for: Analysis of IGH allele content in a sample group of rheumatoid arthritis patients demonstrates unrevealed population heterogeneity
Source: Front Immunol. 2023 Jan 31;14:1073414. doi: 10.3389/fimmu.2023.1073414 (PMC9927645; doi:10.3389/fimmu.2023.1073414)
Supplement: Supplementary file 5 [file Table_3.docx]

**Supplemental Table 3.**

| **Restriction Fragment Length Polymorphism PCR** | |
| --- | --- |
| Hu_VH4-34_RFLP_F | GGACACGGCTGTGTATTACTGTGC |
| Hu_VH4-34_RFLP_R | GCCTACCTGCAGGGAGGT |
| **Targeted genomic validation PCR** | |
| Hu_VH4-34_F | CACCCACATGCAAATCCTCACTTAGG |
| Hu_VH4-34_R | GGCAAAATGAGTCATGCAGGAACTTGTAGG |
| Hu_JH6_F | CTCCGCTTCACCTGGAGCATTCTC |
| Hu_JH6_R | GGCTCAGTTACTCCATCAGACGCACC |
